# Combined Effect of Plasma-Activated Water and Topotecan in Glioblastoma Cells

**DOI:** 10.3390/cancers15194858

**Published:** 2023-10-05

**Authors:** Beatriz Pinheiro Lopes, Liam O’Neill, Paula Bourke, Daniela Boehm

**Affiliations:** 1 School of Chemical and Bioprocess Engineering, University College Dublin, D04 V1W8 Dublin, Ireland; bia.p.lopes.231@gmail.com; 2 Environmental Sustainability and Health Institute and School of Food Science and Environmental Health, Technological University Dublin, D07 H6K8 Dublin, Ireland; paula.bourke@ucd.ie; 3 TheraDep Ltd., QUESTUM Innovation Centre, Limerick Institute of Technology, E91 V329 Clonmel, Ireland; liamoneill@theradep.com; 4 Plasma Research Group, School of Biosystems and Food Engineering, University College Dublin, D04 V1W8 Dublin, Ireland; 5 Conway Institute, University College Dublin, D04 V1W8 Dublin, Ireland

**Keywords:** glioblastoma, anticancer treatment, plasma-activated water, reactive species, topotecan, combination treatments

## Abstract

**Simple Summary:**

New forms of cancer treatment are needed to target resistant cancers or reduce severe side effects associated with some treatments. Cold Atmospheric Plasma (CAP) has demonstrated promising anti-cancer effects in several types of cancer, not only by direct application onto cancer cells, but also by indirect treatment using activation of liquids, such as water. Although the exact mechanism of action and underlying signaling pathways are yet to be discovered, CAP has the potential to be combined with traditional therapies, such as chemotherapy. This study investigates a new approach for treating brain cancer by combining a conventional cancer drug with plasma-activated water, which is produced by exposing water to plasma. The reactive chemical species in these solutions may help to kill the cancer cells and reduce the amount of drug that is needed. Not only were the combined treatments able to interfere with cell metabolism and increase cell death, but they also reduced the long-term cell proliferation. This study provides evidence that indirect CAP-derived approaches could be used in combination with chemotherapeutics for prospective treatment of brain cancer.

**Abstract:**

The increase in cancer diagnoses and cancer deaths, severe side effects of existing treatments and resistance to traditional treatments have generated a need for new anticancer treatments. Glioblastoma multiforme (GBM) is the most common, malignant and aggressive brain cancer. Despite many innovations regarding GBM treatment, the final outcome is still very poor, making it necessary to develop new therapeutic approaches. Cold atmospheric plasma (CAP) as well as plasma-activated liquids (PAL) are being studied as new possible approaches against cancer. The anticancer activity of PAL such as “plasma-activated water” (PAW) is dependent on the reactive chemical compounds present in the solution. Possible combinatory effects with conventional therapies, such as chemotherapeutics, may expand the potential of PAL for cancer treatment. We aim to explore the therapeutic properties of a combination of PAW and topotecan (TPT), an antineoplastic agent with major cytotoxic effects during the S phase of the cell cycle, on a GBM cancer cell line (U-251mg). Combined treatments with PAW and TPT showed a reduction in the metabolic activity and cell mass, an increase in apoptotic cell death and a reduction in the long-term survival. Single applications of PAW+TPT treatments showed a cytotoxic effect in the short term and an antiproliferative effect in the long term, warranting future exploration of combining PAW with chemotherapeutic agents as new therapeutic approaches.

## 1. Introduction

According to the World Health Organization, glioblastoma multiforme (GBM) is considered a grade IV astrocytoma [1], making it the most common and aggressive malignant primary brain tumour in adults. GBM presents a poor prognosis and quality of life, low survival rates (less than 1 year for most patients and only about 5% survival after 5 years) [2,3] and high resistance to chemotherapy due to the blood–brain barrier [4,5]. The current standard therapy includes surgical resection followed by adjuvant radiation and chemotherapy [6]. However, complete remission remains elusive [7], which makes it critical to identify and develop novel technologies that are more accurate and less toxic not only for treatment but also for in vivo diagnosis, prognosis and treatment [4,7].

Topotecan (TPT) is a water-soluble antineoplastic agent, a semi-synthetic analogue to camptothecin and a topoisomerase I (Top I) inhibitor, that interferes with the cell cycle and cellular metabolic profile [8]. Despite having potent antitumour activity with minimal consequences for the normal brain in preclinical models, systemic administration of TPT results in limited and poorly tolerated toxicities without appreciable antitumour effects, which limits its use for GBM cases [9]. Recent studies have shown that local TPT (co-)administration could be implemented using novel therapeutic approaches, such as convection-enhanced delivery, stereotactic injections, implanted reservoirs or intra-arterial delivery [5,8,10].

Recently, cold atmospheric plasma (CAP) as well as plasma-activated liquids (PAL) have emerged as promising non-thermal technologies (operating at atmospheric pressure and near room temperature) and are being studied as new potential cancer treatments [11]. CAP is a partially ionized gas that contains electrons, excited atoms, charged particles, free radicals, reactive oxygen (such as O•, •OH, HO_2_•, OH^–^, O_2_^–^, O^–^, O_2_^+^, O^+,^ O_3_ and H_2_O_2_) and reactive nitrogen (such as N•, N2*, N*, N_2_^+^, N^+^, NO and NO•) species, UV photons and electromagnetic fields [4].

Plasma-activated liquids (PAL), also known as plasma-treated, plasma-functionalized or plasma-conditioned liquids, refer to liquids that have been exposed to a plasma discharge, resulting in the generation and/or dissolution of reactive species in the liquid phase, which can alter biological molecules, induce cellular stress responses or lead to cell death [12,13,14].

The use of plasma-generated solutions (such as water or physiological saline) may allow for better delivery of reactive species [15] to internal body locations and provide therapeutic effects not possible with direct plasma treatment [11]. Some of the most promising application fields of plasma-activated liquids are the plasma-based preparation, optimization or stabilization of mainly liquid pharmaceutical preparations and the support of drug transport across biological barriers [16]. The transfer of plasma species to the aqueous phase can create bioactive solutions that are more easily injected into the bloodstream or administered to difficult-to-reach places, such as internal tumours, thus providing therapeutic effects that would not be possible with direct plasma treatment (due to the dimension of the plasma devices) [16]. Plasma-treated solutions could be safer than direct treatment because they avoid direct tissue exposure to potentially undesirable plasma mechanisms, such as UV radiation, and have benefits such as the possibility for off-site generation and storage. The storage temperature and the composition also have an impact on the stability (during storage) and anticancer effects of plasma-activated solutions [11]. Possible combinatory effects with conventional therapies, such as chemotherapeutics, may expand the potential of PAL for cancer treatment.

GBM remains challenging to treat, and future treatments will probably be based on different combinatorial approaches in order to have the best outcome for patients. The main objective of the present study is to shed light on the potential interaction of PAW with TPT as a new combination therapy for the local treatment of GBM. A clear set of objectives was defined to address: the relation between the RONS composition and the effect in the U-251mg cell line, an assessment of the effect of the individual PAW, TPT or combined treatments on cell survival rate, an evaluation of the effect of the treatments in cell death pathways and its influence on the cell cycle, as well as the cells’ long-term response to individual PAW, TPT or combined treatments.

## 2. Materials and Methods

### 2.1. Experimental Setup for Plasma Generation

Plasma treatment was based on two types of electrical discharges: glow discharge (G) and spark discharge (S) performed using a stainless-steel needle as the high voltage (H.V.) electrode, which was fixed perpendicular to the solution’s surface [17].

**Figure 1:**
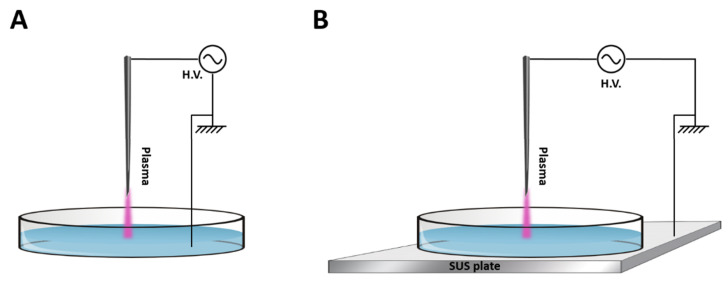
Schematic of air discharges of (**A**) glow discharge and (**B**) spark discharge above water (adapted from [17]).

Both types of discharges were operated in atmospheric air. The maximum output voltage was 20 kV with a variable frequency of 20–65 kHz depending on the plasma load capacitance and a fixed frequency of 25 kHz. Working power used was 16 Watt for glow discharge and 19 Watt for spark discharge. For the generation of PAW, 10 mL of sterile deionised water was added into a plastic petri dish (Sarstedt Ltd., Drinagh, Ireland), which corresponded to a water layer of about 4.2 mm depth. The power supply used for driving plasma discharges was an H.V. half bridge resonant inverter circuit (PVM500, INFORMATION UNLIMITED) [17]. The configuration of each discharge is shown in Figure 1. The main difference between the setups is the connections of the ground electrode. In the glow setup, a thin stainless-steel ground electrode was submerged into the liquid sample contained in the petri dish (Figure 1A). In the spark setup, the petri dish was placed on a stainless-steel plate that was connected to the ground (Figure 1B). The system was operated at atmospheric pressure using atmospheric air, with a fixed frequency of 25 kHz and a distance between the HV needle tip and the liquid surface of 5 mm in all experiments [17].

### 2.2. Plasma-Activated Water Generation and pH Measurements

PAW was generated from 10 mL of deionized water added into a 55 mm internal diameter polystyrene petri dish (Sarstedt Ltd., Drinagh, Ireland) and exposed to the glow plasma discharge (Figure 1A) or spark plasma discharge (Figure 1B) for 5, 10 or 15 min. The pH of PAW was measured by an Orion pH meter (model 420A, Thermo Electron Corporation, Waltham, MA, USA).

### 2.3. Chemical Analysis of Reactive Species in Plasma-Activated Water

#### 2.3.1. Determination of Hydrogen Peroxide (H_2_O_2_) and Reactive Oxygen Species (ROS)

H_2_O_2_ concentrations in PAW were quantified using the titanium oxysulfate (TiOSO_4_, Sigma-Aldrich, Arklow, Ireland) colorimetric method. A total of 100 µL of each sample of PAW was incubated with 10 µL TiOSO_4_ in the dark for ten minutes. Absorbance was read on a spectrophotometric plate reader (ThermoScientific, Waltham, MA, USA) at 405 nm wavelength. A standard curve of known H_2_O_2_ concentrations was included on each plate and used to convert absorbance into H_2_O_2_ concentration [18].

Total oxidative species in PAW were measured using the potassium iodide (KI, Sigma-Aldrich, Arklow, Ireland) colorimetric method. A total of 50 µL of PAW or H_2_O_2_ standard samples were mixed with 50 µL deionized water and 100 µL 1 M potassium iodide (Sigma-Aldrich, Arklow, Ireland), incubated for twenty minutes, and the absorbance was read at 390 nm wavelength [19].

#### 2.3.2. Determination of Nitrite (${NO}_{2}^{-}$) and Nitrate (${NO}_{3}^{-}$)

${NO}_{2}^{-}$ concentrations were determined using Griess reagent (Sigma-Aldrich, Arklow, Ireland). A total of 50 µL of PAW or nitrite standard were incubated with 50 µL of Griess reagent for thirty minutes. Absorbance was read at 548 nm, and the results were compared to a sodium nitrite standard curve.

${NO}_{3}^{-}$ concentrations were determined photometrically by 2,6-dimethyl phenol (DMP) using the Spectroquant^®^ nitrate assay kit (Merck Chemicals, Darmstadt, Germany) adapted to a 96-well format. All the PAW samples were pretreated with sulfamic acid to eliminate nitrite interference. A total of 25 µL of the pretreated PAW sample, 200 µL of reagent A and 25 µL of reagent B were mixed and incubated for twenty minutes. Absorbance was read at 340 nm and the results were compared to a sodium nitrate standard curve [13,14].

### 2.4. Cell Culture of Human Glioblastoma Multiforme Cell Lines

Human glioblastoma multiforme cell lines A172 and U-251mg (formerly known as U-373 MG-CD14) were kindly provided by Prof. Dr. James Curtin, TU Dublin. Cells were cultured in Dulbecco’s Modified Eagle Medium/Nutrient Mixture F-12 Ham (DMEM/F12, Sigma-Aldrich, Arklow, Ireland) supplemented with 10% Fetal Bovine Serum (FBS, Sigma-Aldrich, Arklow, Ireland) and 2 mM L-Glutamine (Sigma-Aldrich, Arklow, Ireland) in a humidified incubator at 37 °C with 5% CO_2_. Cells were routinely subcultured when 80% confluence was reached using 0.25% *w*/*v* Trypsin solution. For each assay, cells at a density of 2.5 × 10^4^ cells/mL were plated in a 96-well (100 μL) or in a 24-well (600 μL) plate and incubated overnight to allow cell adhesion.

### 2.5. Cytotoxicity Evaluation

#### 2.5.1. Single Treatments

Dose–response curves for glow and spark PAW, and topotecan (TPT) (Sigma-Aldrich, Arklow, Ireland) were established. TPT was dissolved in dimethyl sulfoxide (DMSO) (Sigma-Aldrich, Arklow, Ireland) at a final concentration of 1 mM and stored at −20 °C. This stock was subsequently used to make the working standard solutions in media. The highest concentration of DMSO used was a 5 µM final concentration. Existing media were removed from each well and cells were treated with either PAW, TPT or solvent control (DMSO) and incubated for 72 h. No deleterious effects were observed from the control solvent (range of concentrations between 0.02 nM and 5 µM). Dose–response curves were established for the final % (*v*/*v*) of PAW (spark discharge—1 to 10%; glow discharge—5 to 40%), and the final concentration (nM) of TPT (500.00, 250.00, 125.00, 62.50, 31.25, 15.62, 7.81, 3.91, 1.95, 0.98, 0.49, 0.24, 0.12, 0.06, 0.03, 0.015, 0.008, 0.004 and 0.002 for U-251mg cell line and 250.00, 125.00, 62.50, 31.25, 15.62, 7.81, 3.91, 1.95, 0.98, 0.49, 0.24, 0.12, 0.06, 0.03, 0.015, 0.008 and 0.004 for A172 cell line) in each well.

#### 2.5.2. Combined Treatment

Based on individual treatment dose–response curves, different conditions around the individual IC50 value were selected for further combinatorial studies. For the PAW treatments, final volumes of 20% for glow discharge and 5% for spark discharge were chosen, while for the TPT treatment, final concentrations of 0.1 and 0.2 nM were selected.

### 2.6. Cell Viability Assays

#### 2.6.1. Resazurin/Alamar Blue Assay

Cell metabolic activity was analysed using the resazurin assay (Sigma-Aldrich, Arklow, Ireland). This assay is based on the reduction of the oxidized blue dye (resazurin) to a pink dye (resorufin) by metabolically active cells (live cells). After 72 h exposure time, cells were washed once with sterile Phosphate-Buffered Saline (PBS) (Sigma-Aldrich, Arklow, Ireland) and incubated for 2 h at 37 °C with 100 μL of resazurin (final concentration 8 μg/mL) in the cell culture medium. Absorbance was monitored by a Varioskan LUX 3020-666 (ThermoScientific, Waltham, MA, USA) at 570 nm using 600 nm as a reference wavelength. Results are expressed as a percentage of metabolic activity normalized to control cells. Control experiments using untreated water or DMSO at corresponding concentrations showed no effect on cell metabolic activity.

#### 2.6.2. Crystal Violet Staining

Cell mass was assessed by crystal violet colorimetric growth assay. After 72 h exposure time, cells were washed once with PBS, and adherent cells were fixed with 70% methanol (Sigma-Aldrich, Arklow, Ireland) for 1 min, and then stained with 0.2% crystal violet solution (Sigma-Aldrich, Arklow, Ireland) for 10 min. Excess stain was rinsed off with water and plates were left to air-dry overnight. The dye bound to the adherent cells was re-solubilised with 10% acetic acid (Sigma-Aldrich, Arklow, Ireland) and absorbance was measured at 600 nm using a spectrophotometric microplate reader (ThermoScientific, Waltham, MA, USA). Cell mass is expressed as percentage normalized to control cells. Control experiments using untreated water or DMSO at corresponding concentrations showed no effect on cell mass.

### 2.7. Combination Index

To determine the nature of interaction of the combination of PAW and TPT in the U-251mg cell line, the combination index (CI) value was calculated as:

$$CI=(C_{PAW}/I_{PAW})+(C_{TPT}/I_{TPT})$$

In this case, C_PAW_ and C_TPT_ refer to the concentrations of TPT and PAW when in combination treatment, while I_PAW_ and I_TPT_ refer to the concentrations of TPT and PAW in individual treatments that achieved the same effect as the combination. In general, CI = 0.7–0.9 indicates a slight synergism (SS), CI = 0.9–1.1 nearly additive (NA), CI = 1.1–1.2 slight antagonism (SA), CI = 1.2–1.45 moderate antagonism (MA), while CI = 1.45–3.3 refers to antagonism (A) [20].

### 2.8. Evaluation of Cell Death by Flow Cytometry

Cell death was measured after treatment using the Invitrogen™ eBioscience™ Annexin V Apoptosis Detection Kit PE (ThermoFisher, Dublin, Ireland) according to the manufacturer’s instructions. Briefly, after 72 h exposure time, cells grown in a 24-well plate were harvested, centrifuged at 500× *g* for 5 min and washed with PBS. After a second wash with Binding Buffer, cells were resuspended in 100 μL of Binding Buffer and incubated for 15 min at room temperature with 1 μL of fluorochrome-conjugated Annexin V-PE. After washing with Binding Buffer, cells were incubated with 1 μL 7-AAD Viability Staining Solution. Annexin V-PE and 7AAD fluorescence was then measured using the CytoFLEX Flow Cytometer (Beckman Coulter Inc, Indianapolis, IN, USA) with a blue laser (488 nm) using FL-2 and FL-3 detectors. Results are expressed in the percentage of early apoptotic, late apoptotic/necrotic, necrotic and viable cells.

### 2.9. Evaluation of Cell Proliferation and DNA Content by Flow Cytometry

The detection of cell proliferation and DNA content was performed using the baseclick EdU Flow Cytometry Kit (baseclick, Sigma-Aldrich, Arklow, Ireland) according to the manufacturer’s instructions. In brief, after 72 h exposure time, cells were incubated with 10 µM EdU (5-ethynyl-2′-deoxyuridine) for 2 h. Then, cells were harvested, centrifuged at 500× *g* for 5 min, washed with 1% BSA in PBS and fixed with the fixative solution. After a second wash with 1% BSA in PBS, cells were incubated with saponin-based permeabilization buffer in PBS before starting the click reaction. Cells were then washed with saponin-based permeabilization buffer and wash reagent, and incubated with a 10 µg/mL Propidium Iodide (PI)/100 µg/mL RNase solution in PBS (PI/RNase, Sigma-Aldrich, Arklow, Ireland) for 15 min at 4 °C. EdU and PI fluorescence was then measured using the CytoFLEX Flow Cytometer with a blue laser (488 nm) using FL-1 and FL-2 detectors. Data were analysed using CytExpert version 2.4.0.28 software (Beckman Coulter Inc., Indianapolis, IN, USA). For each assay, data of at least 10,000 events were collected.

### 2.10. Evaluation of Cell Survival Clonogenic Assay

To evaluate the ability of a single cell to form a colony, a cell survival assay was performed. After 72 h exposure time to PAW and/or TPT, 500 cells per condition were harvested and plated in 6-well plates in a total volume of 3 mL of complete medium. The medium was renewed after 7 days, and after 14 days, colony formation was assessed by staining with crystal violet solution (as described above in 2.6.2). The survival factor was calculated as follows [21]:

(1) $$Survival\;factor\;\left(\%\right)=\frac{absorbance\;of\;treated\;cells}{absorbance\;of\;control\;cells}\times 100$$

### 2.11. Statistical Analysis

All the experiments were performed at least three independent times. Prism version 8.0.1, (GraphPad Software, San Diego, CA, USA) was used to carry out curve fitting and statistical analysis. Dose–response curves were measured using nonlinear regression. Data are presented as the mean and standard deviation (SD). Multiple comparison analyses were performed using two-way ANOVA with Dunnett post-test for chemical analysis and two-way ANOVA with Tukey’s post-test for the rest of the experiments. Differences were considered to be statistically significant at * *p* < 0.05.

## 3. Results

### 3.1. Chemical Composition of PAW Is Setup-Dependent and Influences the Cytotoxic Effect on U-251mg Cell Line

**Figure 2:**
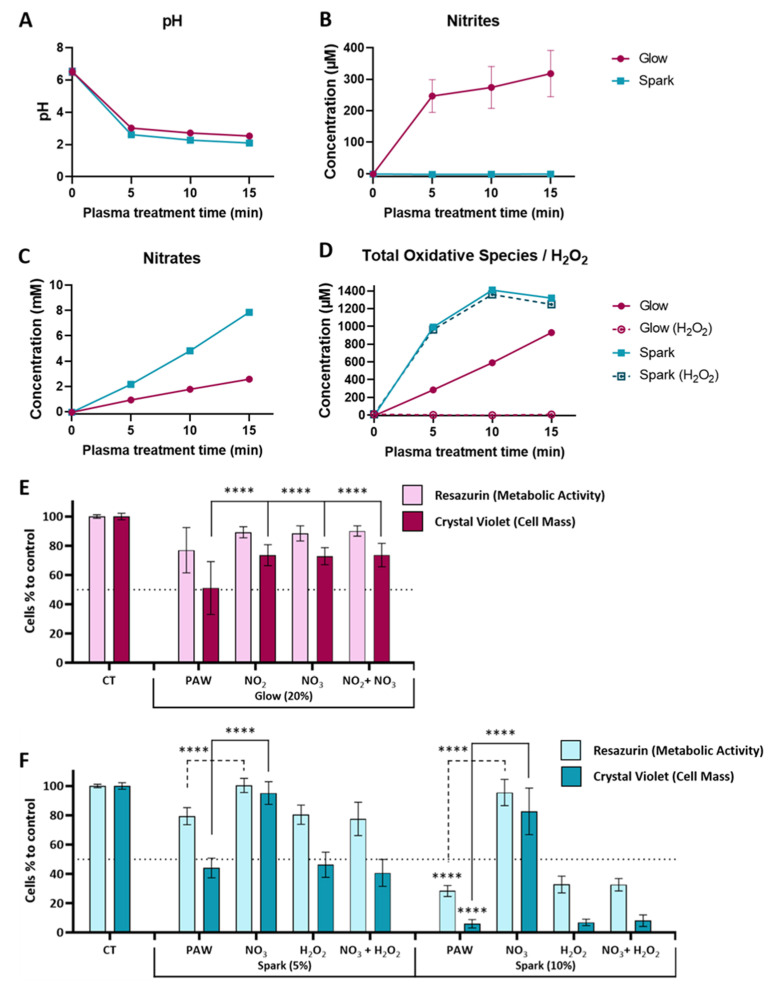
Chemical composition and cytotoxic effect of PAW produced by glow and spark discharge. (**A**) pH value; (**B**) nitrite concentration measured using Griess reagent; (**C**) nitrate concentration measured by Spectroquant^®^ Kit; (**D**) total oxidative species measured by KI (filled lines) and H_2_O_2_ measured by TiOSO_4_ (dotted lines); reduction of the metabolic activity (resazurin) and cell mass (crystal violet) of U-251mg cells by (**E**) glow discharge (15 min) PAW with a dilution of 20% (*v*/*v*), (**F**) spark discharge (5 min) PAW with dilutions of 5% and 10% (*v*/*v*) and solutions mimicking PAW in comparison to control. Results are presented as mean ± SD. Statistical significance is shown for artificial solutions in comparison to PAW (G15, S5 and S10) and represented as **** *p* < 0.0001.

Water samples treated with plasma (glow or spark discharge) for 5, 10 or 15 min in atmospheric air were immediately chemically characterised after treatment. The final pH after 5 min exposure for both types of PAW was acidic, with reduction to levels of 3.02 for glow discharge and 2.62 for spark discharge, remaining relatively stable even with additional treatments of up to 15 min (Figure 2A). Nitrites were only detected in glow discharge with concentrations around 318 µM for a 15 min treatment time (Figure 2B). On the other hand, the nitrate concentrations increased in both plasma discharges over the course of the treatment with an increase from 0.93 to 2.57 mM (5 min to 15 min of treatment time) for glow discharge PAW, and from 2.16 to 7.86 mM for spark discharge PAW (Figure 2C). As previously described for the glow discharge plasma setup [13,14], no presence of H_2_O_2_ was detectable using TiOSO_4_. However, a reaction with KI indicated the presence of other peroxides/oxidative species, with increasing levels up to 930 µM with 15 min of glow discharge (Figure 2D). Conversely, in spark discharge PAW, H_2_O_2_ levels increased over the course of the treatment reaching their maximum/peak (1359 µM) after 10 min and remaining stable after that (Figure 2D). The results from the KI reaction indicate that the oxidative species in spark discharge PAW were almost exclusively H_2_O_2_ since similar concentrations were observed (Figure 2D).

To investigate the influence of the chemical composition of PAW on the cytotoxic effect of those solutions, artificial solutions were prepared to mimic the chemical composition of each PAW. Solutions with individual species and an artificial mixture of both species were prepared and the effect was compared with the specific PAW using a glioblastoma cell line (U-251mg). We selected PAW treated with glow discharge for 15 min (G15) and spark discharge for 5 min (S5) since they presented similar pH values and nitrate levels (Figure 2A,C) and contrasting nitrite and H_2_O_2_ levels. The concentrations used for each species were similar to the ones obtained from the chemical analysis (300 µM for nitrates, 2 mM for nitrates and 1000 µM for H_2_O_2_). For glow treatments, nitrites and nitrates, alone and in combination, were not able to replicate the effect obtained for the PAW (significant difference of PAW compared to nitrites and/or nitrates for cell mass but not metabolic activity), indicating that other oxidative species (not identified) influence the effect of glow discharge PAW on glioblastoma (Figure 2E), while for the spark treatment, H_2_O_2_ alone and in combination with nitrate was able to replicate the effect of PAW on glioblastoma cells, suggesting a critical role for H_2_O_2_ in the cytotoxic effect of spark treatments (Figure 2F).

### 3.2. PAW and TPT Combinations Decrease the Survival Rate of Glioblastoma Cells

**Figure 3:**
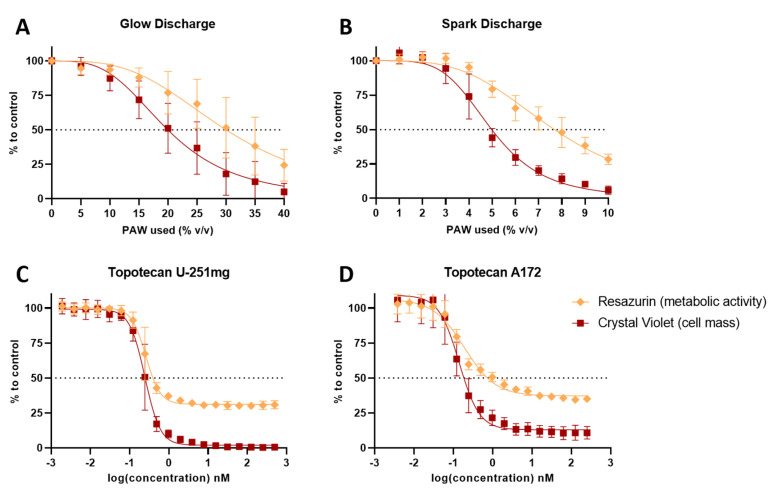
Metabolic activity and cell mass dose–response effect of PAW or TPT individual treatments in glioblastoma cell lines. Dose–response curves were obtained by resazurin and crystal violet assays. IC50 curves of (**A**,**B**) PAW and (**C**) TPT treatments alone in U-251mg cell line as well as (**D**) in A172 cell line. Results are presented as mean ± SD and as comparisons to control.

In order to investigate combinatory effects between PAW and TPT, U-251mg and A172 cell lines were treated with increasing volumes of PAW or increasing concentrations of TPT, and then, the metabolic activity (resazurin) and cell mass (crystal violet) were evaluated (Figure 3). The estimated theoretical IC50 values calculated demonstrated that the glow treatment (PAW by glow discharge for 15 min—hereafter referred to as G15) requires four times more volume to show a similar cytotoxic effect to that of the spark treatment (PAW by spark discharge for 5 min—hereafter referred to as S5) (Table 1). A172 cells were more sensitive to the TPT treatment than U-251mg cells, displaying a lower IC50 value (Table 1).

### 3.3. PAW and TPT Combinations Have an Antiproliferative Effect in Glioblastoma Cells

**Figure 4:**
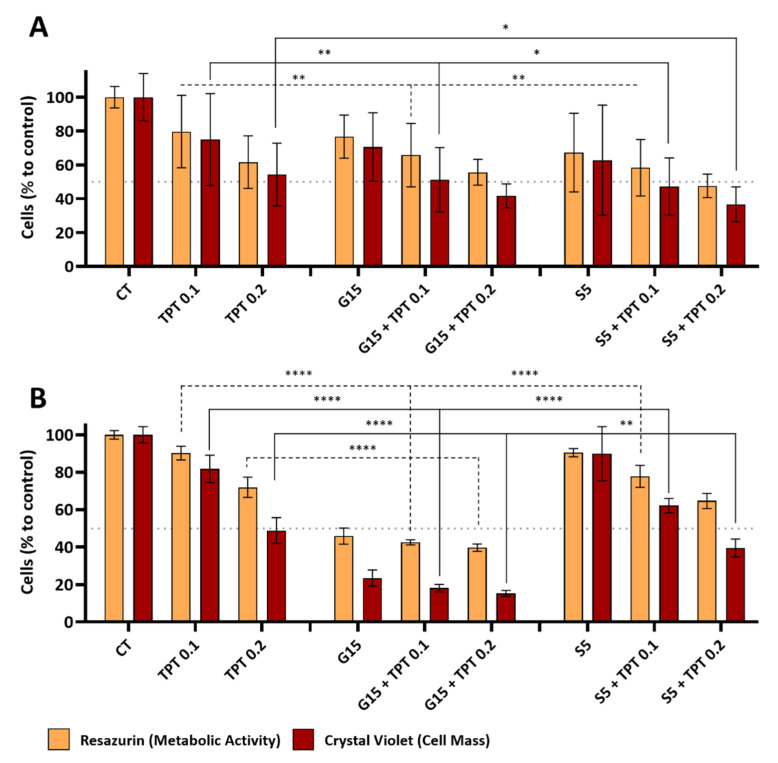
PAW+TPT treatments reduce the metabolic activity and cell mass in glioblastoma cell lines. Resazurin and crystal violet assays were performed after 72 h of treatment with TPT, PAW or PAW+TPT. Quantification of the combinatorial effect was performed in (**A**) U-251mg cell line and in (**B**) A172 cell line. Results are presented as mean ± SD in comparison to control. Statistical significance (dotted line for resazurin and filled line for crystal violet) is shown in comparison to TPT treatment alone (TPT 0.1, TPT 0.2) and represented as: * *p* < 0.05; ** *p* < 0.01; **** *p* < 0.0001.

For the combinatory effects of both PAW and TPT, we selected 20% (*v*/*v*) for the glow treatment (G15), 5% (*v*/*v*) for the spark treatment (S5) and 0.1 and 0.2 nM for the TPT treatments (TPT 0.1 and TPT 0.2). The metabolic activity and cell mass content in the U-251mg and A172 cell lines were evaluated after 72 h of treatment with PAW, TPT and PAW+TPT. For the U-251mg cell line, both metabolic activity and cell mass decreased with TPT 0.1 nM (79.65 ± 21.44% and 74.95 ± 27.17%) and 0.2 nM (61.60 ± 15.57% and 54.25 ± 18.60%), respectively (Figure 4A). The same trend was observed for G15 and S5 alone. For treatments only with PAW, the metabolic activity was decreased (76.66 ± 12.75%) for G15 and (67.22 ± 23.19%) for S5, as well as the cell mass (70.54 ± 20.20%) for G15 and (62.73 ± 32.54%) for S5. The effects observed after combinatorial treatments of G15 or S5 with TPT 0.1 nM were significantly enhanced (*p* < 0.05) when compared to the drug alone (65.75 ± 18.68% and 51.17 ± 19.02%) for G15+TPT 0.1 and (58.29 ± 16.68% and 47.21 ± 16.87%) for S5+TPT 0.1. Combination treatments with TPT 0.2 nM were similar or slightly stronger compared to treatment with only TPT 0.2 nM, with decreases in metabolic activity and cell mass (55.63 ± 7.67% and 41.70 ± 7.07%) with G15+TPT 0.2, and (47.54 ± 6.94% and 36.68 ± 10.28%) with S5+TPT 0.2, respectively.

The single treatment with TPT 0.1 nM (90.25 ± 3.68% and 81.77 ± 7.34%) and TPT 0.2 nM (71.98 ± 5.40% and 48.89 ± 6.85%) decreased the metabolic activity and cell mass, respectively, of A172 cell line (Figure 4B). The single treatment with G15 decreased the metabolic activity to half (45.88 ± 4.30%) and the cell mass to a quarter (23.53 ± 4.30%) when compared to the control. Combination treatments between G15 and TPT 0.1 nM or 0.2 nM achieved a significantly higher reduction (*p* < 0.0001) than the drug treatments alone but maintained levels similar to the treatment with only G15 for metabolic activity (42.54 ± 1.41% (G15+TPT 0.1) and 39.72 ± 1.94% (G15+TPT 0.2)) and for cell mass (18.13 ± 1.94% and 15.30 ± 1.56%), respectively. Treatment only with S5 did not reveal a big effect (90.47 ± 2.20% (metabolic activity) and 89.90 ± 14.47% (cell mass)), but in combination with TPT 0.1 nM and 0.2 nM, that effect was more evident (77.76 ± 5.86% and 62.19 ± 3.81% for 0.1 nM; 64.69 ± 4.06% and 39.54 ± 4.77% for 0.2 nM) and significantly enhanced compared to treatments with only TPT at 0.1 nM and 0.2 nM (*p*-values < 0.01 with the exception of the metabolic activity at S5+TPT 0.2) (Figure 4B).

The combination index (CI) calculated for the PAW+TPT combinations varied between “Nearly Additive” to “Antagonism” (Table 2). As the values are closer to the lower limit of the “Antagonism” definition (CI = 1.45–3.3) rather than the higher limit, further analysis was performed for a better understanding of the effects of the combination treatments.

### 3.4. Apoptosis Is the Main Pathway of PAW- and PAW+TPT-Induced Cell Death

**Figure 5:**
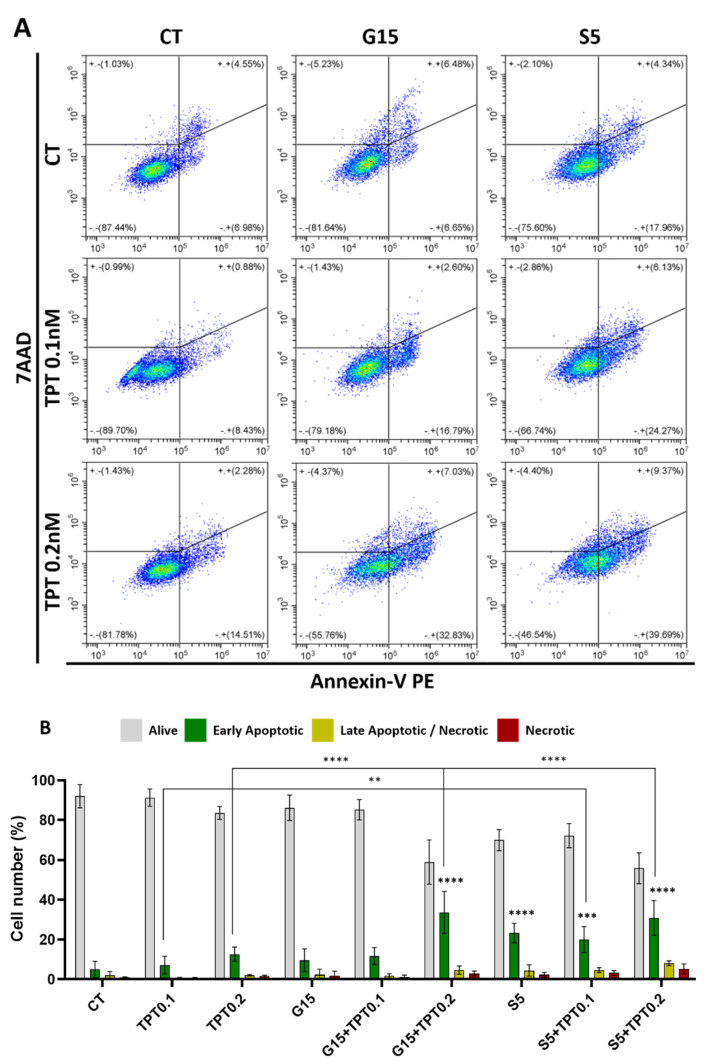
PAW+TPT combination treatment induces apoptosis in U-251mg cells. Apoptosis of U-251mg cells was evaluated by flow cytometry (Annexin V/7AAD) after 72 h incubation with PAW, TPT and PAW+TPT. (**A**) Representative flow cytometry plots and (**B**) quantification of each type of cell death in single and combinatorial treatment. Results are presented as mean ± SD. Statistical significance is shown in comparison to drug treatment alone (TPT 0.1, TPT 0.2) and represented as: ** *p* < 0.01; *** *p* < 0.001; **** *p* < 0.0001.

Flow cytometry results from Annexin V/7AAD staining revealed that PAW and PAW+TPT treatments induced apoptosis in U-251mg cells, especially in PAW+TPT combinations with higher concentrations of TPT (Figure 5A). A shift from the Annexin V negative to the Annexin V positive quadrant can be observed, demonstrating that cells are starting to present markers of the early apoptosis pathway (phosphatidylserine is exposed on the external leaflet of the plasma membrane).

As seen in Figure 5B, the quantification of apoptotic cells showed that in normal conditions, 92.01 ± 5.83% of cells were alive and only 4.97 ± 4.11% were undergoing early apoptosis. Those levels were slightly augmented with TPT 0.1 nM (7.19 ± 4.41%); TPT 0.2 nM (12.63 ± 3.56%); G15 (9.62 ± 5.72%); and G15+TPT 0.1 (11.72 ± 4.27%). Apoptotic cell levels were strongly increased with G15+TPT 0.2 (33.58 ± 10.62%); S5 (23.26 ± 4.86%); S5+TPT 0.1 (19.97 ± 6.47%); and S5+TPT 0.2 (30.81 ± 8.78%). Other mechanisms of cell death such as necrosis (7AAD positive/Annexin V negative cells) only showed minimal occurrence, in line with the concept that cells undergo controlled cell death upon PAW+TPT treatments. 

### 3.5. Combinatorial Treatments of PAW and TPT Impact Cell Cycle

**Figure 6:**
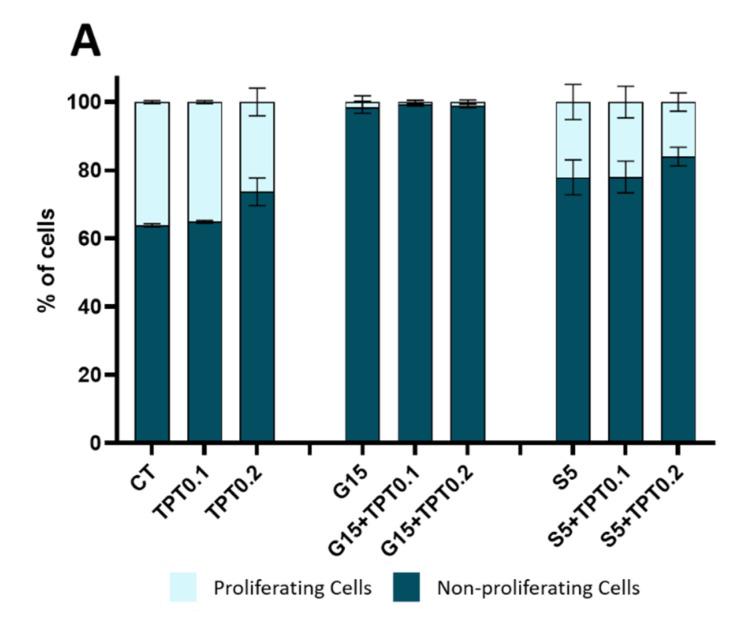

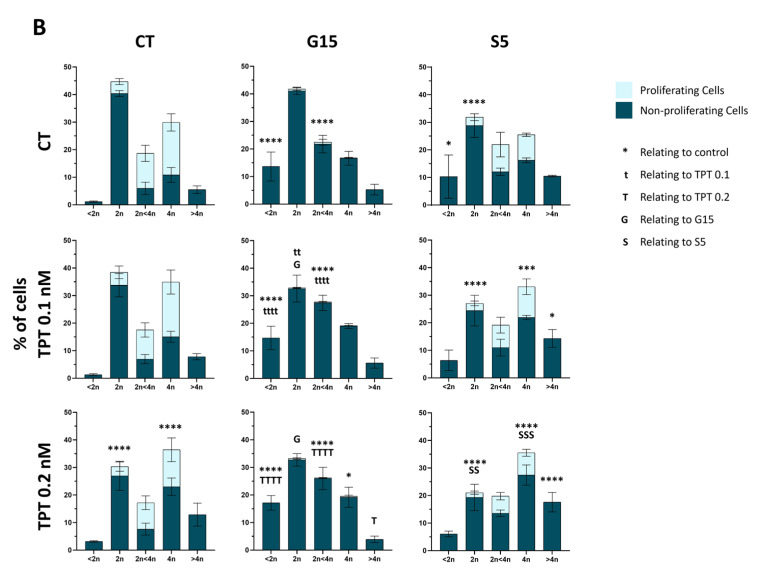
PAW+TPT combination treatment decreases cell proliferation, possibly by promoting cell cycle arrest. U-251mg cells were analysed by flow cytometry (EdU/PI) after 72 h incubation either with PAW, TPT or PAW+TPT. (**A**) Quantification of proliferating and non-proliferating cells based on EdU incorporation. (**B**) Quantification of the different DNA content of the cells based on staining with PI. Results are presented as mean ± SD. Statistical significance analysed relative to the control for non-proliferating cells and represented as: * *p* < 0.05; ** *p* < 0.01; *** *p* < 0.001; **** *p* < 0.0001 and for combination treatments relative to the corresponding single treatments with TPT 0.1 nm (t), TPT 0.2 nM (T), G15 (G) or S5 (S).

The quantification of proliferating cells, as pictured in Figure 6A, demonstrated that in control conditions, glioblastoma cells present 36.12 ± 0.45% of proliferating cells. Upon treatment with TPT at 0.1 nM, the number of proliferating cells was comparable with the control condition (35.12 ± 0.43%), while a slight reduction was observed for treatment with 0.2 nM of TPT (26.31 ± 4.06%). On the other hand, treatment with G15 alone or in combination with TPT 0.1 and 0.2 nM totally reduced the percentage of proliferating cells (1.54 ± 1.78%; 0.69 ± 0.50% and 1.12 ± 0.58% respectively). The treatments with S5 and S5+TPT showed a small reduction in the number of proliferating cells (22.12 ± 5.14%; 21.95 ± 4.67% and 16.01 ± 2.69%), pointing to a delayed cell cycle progression instead of cell cycle blockade.

Moreover, we observed significant changes in the DNA content profile in all treatments (Figure 6B). Non-treated cells (control) showed a normal cell cycle distribution with about 45% of cells showing a DNA content of 2 n (suggestive of G1/G0 phase), around 20% containing 2 < 4 n (S phase) and 30% at 4 n (G2/M) and the remaining cells at <2 n or >4 n. A decrease in cells with a DNA content of 2 n was observed upon TPT and spark PAW individual treatments (30.31 ± 6.73% for TPT 0.2 nM and 31.84 ± 3.91% for S5), and upon combination with TPT 0.2 nM (33.21 ± 2.08% for G15 and 21.03 ± 4.61% for S5) when compared to the control (44.71 ± 0.18%). In addition, the percentage of cells with a DNA content of 4 n and higher DNA content (≥4 n) increased with the TPT and spark individual and S5+TPT 0.1 nM or 0.2 nM treatments (49.31 ± 6.10% for TPT 0.2 nM, 35.94 ± 0.45% for S5, 47.43 ± 0.92% for S5+TPT 0.1, and 53.12 ± 5.43% for S5+TPT 0.2) when compared to the control (35.43 ± 4.10%). As such, these results point to cell cycle arrest in the late S phase or an inability to progress through mitosis in cells treated with TPT, S5 and S5+TPT.

On the other hand, the treatments with G15 and G15+TPT showed a pronounced increase in the sub-2 n population, indicative of higher cell death in these samples. The treatment conditions also showed a reduction in cells in the 2 n and 4 n populations compared to the control, while the percentage of cells with 2 < 4 n slightly increased. Since all G15-treated cells lost the ability to proliferate after 72 h, the profiles may result from a combination of responses: the inability to progress through the cell cycle, along with differences in the susceptibility of the different cell populations to the cytotoxic effects of the treatment with G15 and G15+TPT.

### 3.6. PAW and TPT Combination Treatments Have a Long-Term Antiproliferative Effect

**Figure 7:**
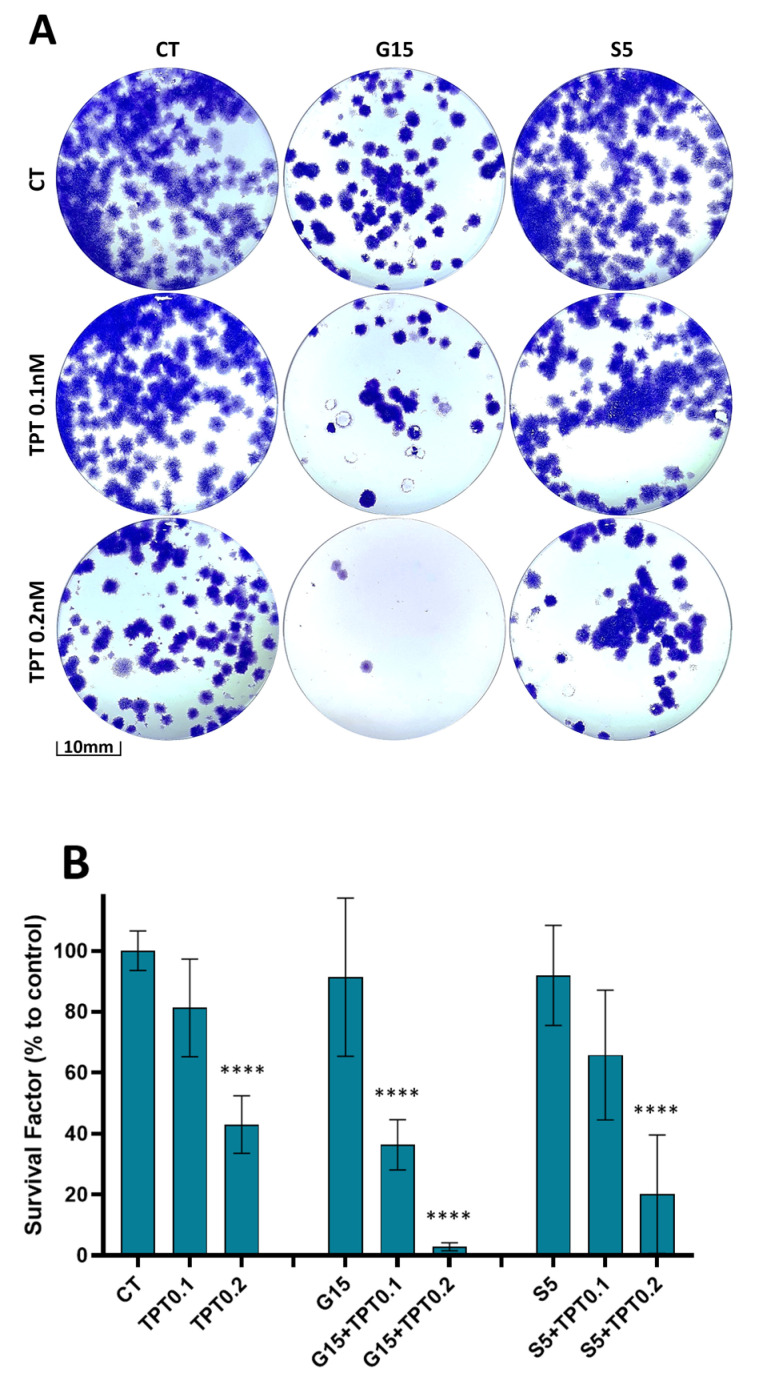
PAW+TPT combination treatment decreases long-term survival. U-251mg cells were treated for 72 h either with PAW, TPT or PAW+TPT. Colony formation was evaluated after 14 days. (**A**) Representative images of the colonies formed 14 days after the end of treatments; (**B**) quantification of cell mass in relation to control. Results are presented as mean ± SD and as comparison to control. Statistical significance is represented as: **** *p* < 0.0001.

In addition to the short-term antiproliferative effect, we evaluated the effect on long-term survival after treatment when cells were recovered in the PAW or TPT free medium. The clonogenic assay revealed a decrease in colony formation, especially when the combination treatment was applied (Figure 7A), and the total cell mass was reduced upon treatment with TPT 0.2 nM (43.02 ± 9.45%); G15+TPT 0.1 nM (36.34 ± 8.26%); G15+TPT 0.2 nM (2.86 ± 1.34%); S5+TPT 0.1 nM (65.78 ± 21.28%); and S5+TPT 0.2 nM (20.12 ± 19.40%) when compared to the control (Figure 7B). Overall, the cells that survived the combinatorial treatments lost their proliferation ability and were not able to form new colonies (especially in combination with the glow PAW treatment). Thus, these results indicate that the surviving cells were blocked in cell cycle progression, and that this blockage seems to be irreversible for the cells treated with the glow PAW in particular.

## 4. Discussion

Recently, the use of the cytotoxic properties of CAP have been recognized as an emerging and promising technique for cancer treatment with a potential application in several cancer types including glioblastoma, melanoma, breast cancer, cervical cancer or prostate cancer [22]. Due to its characteristics, CAP could be used as a replacement for conventional therapies or in combination with conventional therapies to produce a synergistic effect [23]. However, standardization is still problematic since it is dependent on the discharge parameters, the composition of the feed gases used and the duration of plasma administration [24].

PAL has also been studied as an alternative for the direct treatment since it can elicit similar cellular responses and has the potential to be used as a drug (with parameters for drug treatments well established, which may facilitate PAL development/approval) [22]. The concentration of RONS depends on the type of CAP device, the treatment time, the biochemical composition of the solution prepared and the protocol used to deliver RONS to cancer cells [25].

It is known that in the presence of air, short-lived reactive nitrogen species such as (NO or ONOO^−^) are formed in the liquid phase, which subsequently react in water to form acids, affecting the pH of PAL and dissociating to nitrite (${NO}_{2}^{-}$) or nitrate (${NO}_{3}^{-}$) ions. Results for glow PAW suggested that other oxidative species could also be involved in the cytotoxic effect. ${NO}_{2}^{-}$ and ${NO}_{3}^{-}$ do not affect cell viability in the U-251mg cell line, while equivalent concentrations of H_2_O_2_ induced a similar cytotoxic effect to that of spark PAW. In general, ROS induce oxidative stress in cells, activating redox responses [26]. The superoxide anion radical, which is produced by plasma and/or as a cellular product, can react with nitric oxide, generating peroxynitrite, which contributes to lipid oxidation increasing the permeability and fluidity of the membrane [27]. A lower content of cholesterol in the plasma membrane of the cancer cells also decreases the membrane stiffness and may lead to a higher lipid peroxidation, the formation of pores, increased oxidative stress and the induction of apoptosis. A higher expression of aquaporin in cytoplasmic membranes speeds up the absorption of H_2_O_2_ and other small molecules, leading to DNA damage and cell death [28]. The effects of RNS on biological systems are mainly associated with nitrosylation or nitrosation, which modulate different cellular signalling pathways [26]. Understanding the composition and how to modulate and control these solutions can improve the therapeutic approach and represent an alternative for direct treatment with easier internal application [29,30].

The suitability of TPT as a new approach for glioblastoma treatment is being debated [31]. Some authors have suggested that TPT can cross the blood–brain barrier (BBB) [32], while pharmacokinetic data and the high molecular weight suggested that TPT should barely cross the BBB [33]. In order to achieve a cytotoxic concentration in the brain, the systemic dose required triggered extensive systemic disease in human patients [34,35]. Despite these limitations, TPT is being explored as a local therapy for glioblastoma with convection-enhanced delivery [31,33,36]. Our study demonstrated that TPT is able to reduce the metabolic activity and cell mass in two different glioblastoma cell lines (U-251mg and A172) and may be a good candidate for local combined therapy for glioblastoma treatment since only a small percentage of glial cells undergo division and at a slower rate than cancer cells [35].

Although both cell lines (U-251mg and A172) are considered glioblastoma multiforme, they present differences relating to origin, morphology, tumorigenicity and metabolism [37,38], which can influence the effects of the combined treatments [39]. In fact, our results show that the glow PAW treatment (alone and in combination) is more efficient in A172 cells. The spark treatment was only efficient in combination with TPT 0.2 nM. On the other hand, U-251mg cells were more susceptible to TPT combinations irrespective of the discharge source of the PAW generation. These results are reported for the first time here and more work is needed to understand the distinct responses between the cell lines.

It is known that camptothecins, such as TPT, can enter the cells and target Topoisomerase I and also block ribosome formation within minutes of exposure, during the S phase of the cell cycle [35]. Throughout the DNA replication of the S phase, Top I binds to DNA in a momentary cleavable complex and creates grooves in the DNA to reduce torsional stress. After that, the enzyme is released enabling the re-connection of the new strand [40]. TPT blocks the re-connection of these single-strand breaks by binding to the Top I-DNA complex. This binding is reversible but slows down the re-ligation activity of Top I, leading to the overlap of the replication and transcription complexes, thereby generating irreversible Top I covalent complexes with DNA, DNA double-strand breakage and replication arrest, resulting in apoptosis [41].

Despite the general agreement about the RONS contribution, the exact mechanism of action of PAW is still not fully understood. It is accepted that CAP-derived RONS can penetrate the cell membrane, leading to increased levels of intracellular RONS. This increment may induce DNA damage, impair cell division and migration, modulate gene expression and activate apoptosis. It can also induce the activation of the immune system or enhance the effectiveness of chemotherapeutic drugs [25].

In the current study, most of the combinations showed a higher efficacy in reducing cell mass and/or metabolic activity than the individual treatments, particularly at the lower TPT concentration of 0.1 nM, even though the combined effect was not a sum of the individual effects and the combination index (CI) values indicated that all the combined approaches had a slight to moderate antagonist effect after 72 h of treatment. This could be caused, in part, by an overlap of two different mechanisms of action that promote DNA double-strand breakage and the activation of apoptosis, albeit through different pathways. As mentioned before, it is accepted that the therapeutic effects of TPT are due to interference with DNA replication and transcription leading to DNA damage, while PAW-derived RONS can also induce DNA damage and modulate gene expression. Despite some antagonistic action, a combination treatment may nonetheless prove beneficial as it could reduce the drug concentration needed as suggested by enhanced effects achieved by PAW combinations with a lower drug concentration in particular, and could therefore potentially reduce systemic toxicity and side effects.

Similar results were found for GBM when using a combination of CAP and temozolomide (TMZ), an oncology drug used for GBM treatment, supporting the concept that CAP technology can be a suitable candidate for combination therapy with chemotherapeutic drugs [42,43,44]. Combined treatments between CAP and TMZ decreased the cell viability, inducing G2/M cell cycle arrest (accumulation of cells in G2/M phase and a delay in the cell cycle progression), increasing DNA damage and genomic alteration, increasing cell surface integrin expression and reducing cell migration. It was shown that different CAP devices and combination approaches provided analogous outcomes in in vitro studies [43], using 2D models [42] or spheroids [44], and even in in vivo studies [43].

One of the hallmarks of cancer is the ability to evade programmed cell death, allowing cancer cells to expand in number, survive longer, accumulate mutations and evade current therapies. Flow cytometry results showed that combinations with glow PAW treatment tend to trigger the apoptotic pathway in a lower percentage than the combinations with spark PAW treatment. This can be a result of the H_2_O_2_ content present in spark but not glow PAW. It is known that H_2_O_2_ can not only regulate the cell growth and proliferation but also modulate apoptosis or autophagy, due to damage to cellular proteins, lipids and nucleic acids [45]. Moreover, glow PAW treatments (individual or in combination) seemed to promote an almost complete arrest of the cell cycle, indicated by the insignificant levels of proliferating cells after 72 h of treatments. TPT and spark treatments (individual and in combination) showed a similar effect, with some reduction in the proliferating cells, with higher levels of cells blocked at the late S phase, pointing to an impact on cell cycle progression.

The clonogenic capability reflects the ability of a single cell to undergo unlimited division and grow into a colony, which makes it possible to determine the long-term effectiveness of the treatments. Regarding the clonogenicity of glioblastoma cells, all combined approaches were able to successfully reduce the clonogenic capacity of the cells, in line with the reduction in apoptosis and cell arrest results. These results are of particular importance since they demonstrate that the combined treatments are not only cytotoxic to cancer cells, but also affect the reproductive ability of the resistant cells. Cancer stem cells (CSCs) are a subpopulation of cells that can self-renew and are responsible for tumour growth, maintenance and recurrence. CSCs can also contribute to tumour cell heterogeneity and play a major role in drug resistance to chemotherapy and radiotherapy [25]. The survival of CSCs is associated with enhanced antioxidant machinery due to high levels of antioxidant enzymes and the upregulation of antioxidant genes, augmented DNA repair capacity and activation of survival pathways due to the capability of unlimited proliferation [46]. The effects of plasma-generated RONS on CSC are not yet well understood, but it is conceivable that they might be reliant on the specific tumour type, stage and treatment regimen [25].

The results presented in this paper are the first reported for combination treatments between PAW and TPT, and show parallels with other authors’ work where similar results were found in different types of cancer, either with diverse plasma approaches (direct or indirect treatment) or in combination with other oncology drugs: after exposure to PAM or CAP, the survival factor of retinoblastoma cells was highly decreased [21]; the combined treatment of CAP and doxorubicin led to a synergistic effect inhibiting colony formation of mouse melanoma cells [47]; and the combination treatments of plasma-conditioned media and doxorubicin significantly reduced the clonogenic ability in a prostate cancer model [48].

## 5. Conclusions

Overall, our results demonstrate that the reactive species generated by CAP regulate the cytotoxic potential of PAW and that the RONS concentrations depend on the plasma process parameters. The results reveal that combined treatments between PAW+TPT are able to reduce the metabolic activity and cell mass, as well as increase apoptotic cell death in glioblastoma cells. The results presented here also show an increased number of cells with higher DNA content (theoretically, cells in the S phase and G2/M of the cell cycle), a reduction in long-term survival and the inhibition of cell growth with the different combined approaches. On the one hand, combined treatments with PAW+TPT indicate a cytotoxic effect in the short term (effects visible after 72 h). On the other hand, PAW+TPT treatments point to an antiproliferative effect in long-term survival (effects still visible after 14 days).

Nevertheless, the molecular mechanisms of the combined anticancer effects of PAW and TPT remain unclear, and they raise a lot of questions for future exploration, in particular, in relation to elucidating the distinct effects in short- and long-term responses: What specific cellular pathways are involved and is the combined treatment primarily affecting the cell membrane, mitochondria or DNA? Are proteins or genes being altered after the combined treatment? To what extent is the cell to cell communication being affected? Further analyses of distinct cell subpopulations, particularly in relation to their cell cycle stage and their sensitivity to the treatment, may provide further valuable insights. Despite the need for further detailed exploration, this combined approach between PAW and TPT reveals itself as a potential therapy against glioblastoma, which may enable a reduction in drug concentrations and reduce systemic toxicity.

## Figures and Tables

**Table 1:** IC50 values for individual treatments obtained in glioblastoma cell lines.

Cell Line	IC50 Value	Resazurin	Crystal Violet
U-251mg	**Glow (G15)**	30.03%	19.98%
U-251mg	**Spark (S5)**	7.69%	4.98%
U-251mg	**Topotecan**	0.2579 nM	0.2453 nM
A172	**Topotecan**	0.1903 nM	0.1401 nM

Glow (PAW by glow discharge for 15 min); spark (PAW by spark discharge for 5 min).

**Table 2:** Combination index for each combinatorial approach obtained in U-251mg cell line.

Combination Index		Resazurin		Crystal Violet
**G15+TPT 0.1**	1.1696	Slight Antagonism	1.4212	Moderate Antagonism
**G15+TPT 0.2**	1.3157	Moderate Antagonism	1.5627	Antagonism
**S5+TPT 0.1**	1.0498	Nearly Additive	1.3801	Moderate Antagonism
**S5+TPT 0.2**	1.1175	Slight Antagonism	1.5116	Antagonism

G15: PAW by glow discharge for 15 min; S5: PAW by spark discharge for 5 min; TPT: topotecan.

## Data Availability

All data generated or analysed during this study are included in this published article and are available from the corresponding author upon reasonable request.
